# Web‐based cognitive training for breast cancer survivors with cognitive complaints—a randomized controlled trial

**DOI:** 10.1002/pon.4058

**Published:** 2016-01-13

**Authors:** MF Damholdt, M Mehlsen, MS O'Toole, RK Andreasen, AD Pedersen, R Zachariae

**Affiliations:** ^1^ Unit for Psychooncology and Health Psychology, Department of Oncology, Aarhus University Hospital and Department of Psychology and Behavioural ScienceAarhus UniversityAarhusDenmark; ^2^ StoubyDenmark

**Keywords:** web‐based cognitive training, breast cancer survivors, chemo brain, cancer, oncology

## Abstract

**Background:**

Cognitive complaints are common amongst breast cancer survivors, and no standard treatment exists. The present study evaluates whether web‐based cognitive training can alleviate subjectively reported and objectively assessed cognitive complaints in a sample of breast cancer survivors. The primary and secondary outcomes were an objective measure of working memory and a measure of perceived cognitive functioning. Additional outcomes were neuropsychological tests of memory, executive function, working memory and questionnaire‐based assessment of anxiety, depression and somatization.

**Methods:**

A total of 157 female breast cancer survivors were recruited from an existing cohort and through announcements in open access cancer‐related Internet fora and randomly allocated to either web‐based cognitive training (eCogT) with telephone support (*n* = 94) or a waitlist control (WLC) condition (*n* = 63). eCogT encompassed 30 training sessions over 6 weeks. Neuropsychological assessments were undertaken over the telephone, and questionnaire data was collected online. Data was collected at baseline, post‐intervention and at 5‐month follow‐up.

**Results:**

Mixed linear models revealed no statistically significant change in primary or secondary outcome at follow‐up in either group. Statistically significant improvements (*p* 0.040–0.043) were found in the eCogT group for verbal learning and on a working memory test.

**Conclusions:**

Web‐based cognitive training did not result in improvements of the primary or secondary outcome. Improved performance was observed on verbal learning and working memory. These effects were observed at 5‐month follow‐up, indicating long‐term effects of training. The intervention may be applied in a clinical setting at low cost and without risk of adverse effects.© 2016 The Authors Psycho‐Oncology Published by John Wiley & Sons Ltd.

## Background

Cognitive complaints associated with cancer treatment are common and reported by 17–70% of breast cancer survivors 1. Several neuropathological underpinnings for the cognitive impairments have been suggested, including disrupted fronto‐striatal networks and reduced frontal‐occipital and parietal white matter integrity, which have been correlated to impairments in attention, working memory, executive function and memory 1, 2. The cognitive sequelae may be related to cancer treatments: that is, chemotherapy, radiotherapy or hormonal therapies or a consequence of disease‐ and treatment‐related factors, for example, fatigue, metabolic abnormalities and distress 2. The experience of cognitive impairment may prolong cancer‐related disability, reduce quality of life and have detrimental effects on activities of daily living 3, 4, 5.

Notwithstanding uncertainties regarding the nature of cognitive impairment amongst breast cancer survivors, the challenge of developing rehabilitative strategies remains. Pharmacological therapies, for example, modafinil and methylphenidate, appear largely unsuccessful at targeting these impairments and/or are associated with considerable side effects 6, 7. Instead, cognitive interventions may hold promise, as studies have shown positive results on objectively assessed cognition and subjective cognitive complaints 1. However, most studies examine in‐clinic interventions guided by health care professionals in small samples 1, thereby increasing costs and limiting dissemination. These challenges may be overcome by web‐based interventions that have shown beneficial effects on cognition in healthy elderly 8, although not consistently 9. Results of the so far only web‐based cognitive training program for breast cancer survivors indicate improved post‐training cognitive performance 10. This randomized waitlist‐controlled study included 41 breast cancer survivors, whereof 21 received web‐based cognitive training of executive function four times a week for 12 weeks. Improvements in verbal fluency, processing speed and cognitive flexibility were reported alongside improved subjective cognitive functions. Unfortunately, the durability of effects is unknown because of lack of long‐term follow‐up. Further research is clearly needed. We therefore examined effects of web‐based cognitive training in a large sample of breast cancer survivors, applying a broad cognitive training program with baseline and short‐ and long‐term telephone‐based neuropsychological assessments. We hypothesized that breast cancer survivors engaging in web‐based cognitive training would (i) improve their working memory measured by the Paced Auditory Serial Addition Test (PASAT, primary outcome), (ii) report improved subjectively experienced cognition, and (iii) improve on other measures of cognition.

## Methods

### Participants

**Figure 1:**
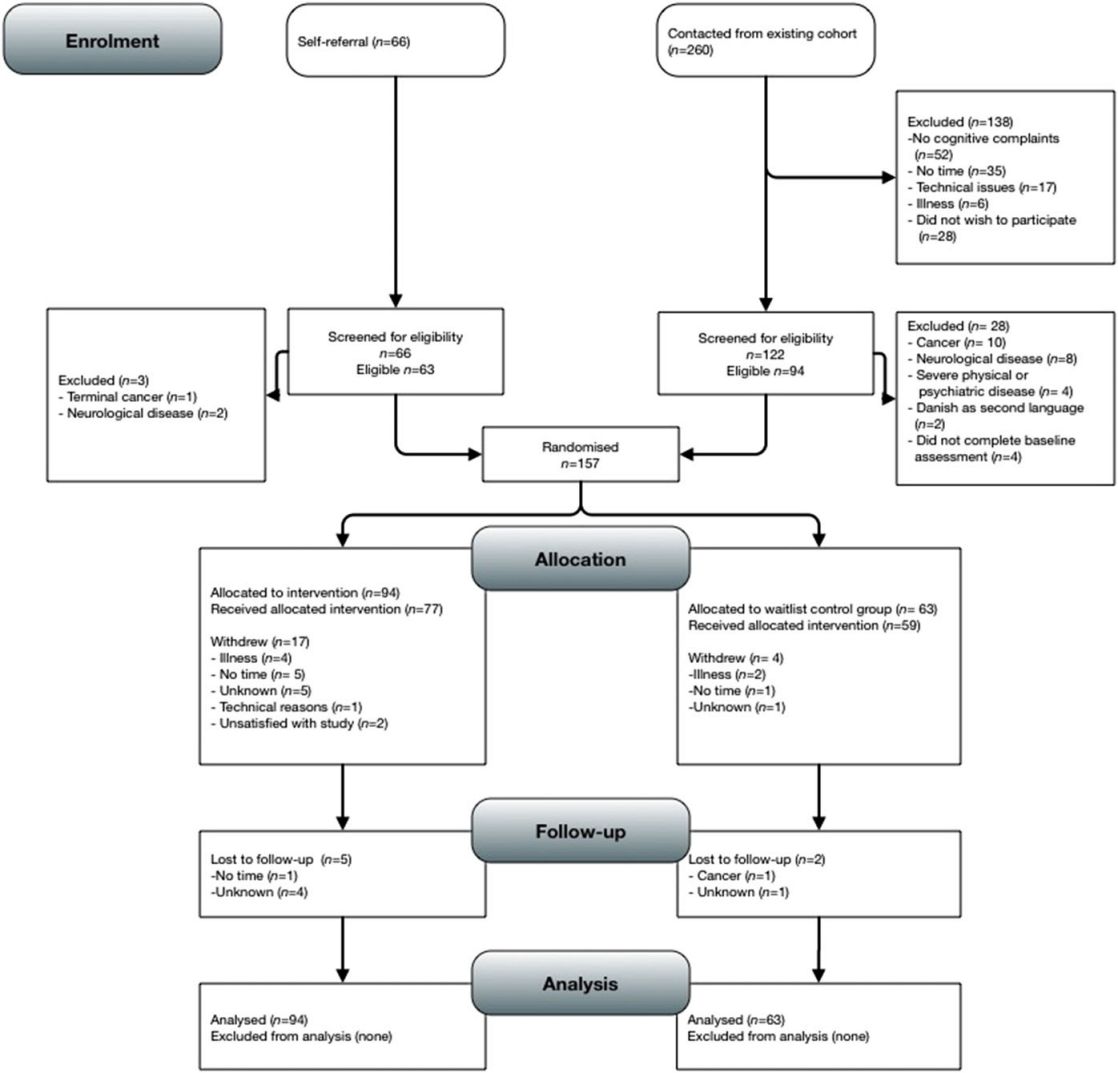
Participant flowchart

Most participants were drawn from an existing cohort of 682 women treated for breast cancer at Aarhus University Hospital who participated in a survey in 2011. Women scoring above the sample median (≥27) on the Cognitive Failures Questionnaire 11 were invited to participate (*n* = 260). An additional 66 women were recruited through announcements on patient association websites and fora for breast cancer and the oncology department, Aarhus University Hospital (refer to Figure 1).

Inclusion criteria were (1) history of breast cancer, (2) subjective complaints of cognitive impairment, and (3) computer and Internet access. Exclusion criteria were (1) head trauma with loss of consciousness (≥30 min), (2) neurological disease, (3) severe physical or psychological disease (e.g. major depression, cerebrovascular disease), (4) alcoholism or drug abuse, (5) non‐fluency in Danish, (6) breast cancer recurrence, (7) a second cancer, (8) severe hearing loss, and (9) inability to read print on a computer screen.

Sample size estimation was based on a normative sample 12 as no data on cognitive training for breast cancer survivors for our primary outcome were available. A sample size of 160 (2:3 randomization = 96:64) was deemed sufficient to detect a mean change score of 3.7 points (Cohen's *d* = 0.35) on the primary outcome PASAT (mean = 48.7, SD = 10.7) with a statistical power of 80% and an alpha of 0.05 12. A 2:3 randomization to the intervention group was chosen to accommodate an expected higher dropout rate among participants in the control group. A researcher uninvolved in the cognitive training generated the randomization sequence in block lengths of ten using a computer‐based procedure 13.

Enrolment began March 2013 and ended December 2014. Enrolment was extended because of recruitment difficulties and suspended during long public holidays.

### Procedure

The study was conducted in accordance with the Helsinki Declaration and preregistered at ClinicalTrials.gov (ID: NCT01866813). Approval was obtained from the local ethics committee and reporting adheres to the CONSORT EHEALTH statement 14.

The women received information about the study by mail and telephone, before written informed consent was obtained. Eligibility screening and neuropsychological assessments were completed over the telephone. This facilitated nationwide distribution, reduced costs and increased completion. Thereafter, personal links to baseline questionnaires were emailed to the participants. Questionnaires were delivered via Qualtrics Software© (2013; Qualtrics, Provo, UT, USA). After baseline assessments, participants were randomized to (a) an intervention group offered web‐based cognitive training (eCogT) or (b) a waitlist control (WLC) group. The project coordinator, who was blinded to baseline performance, undertook participant allocation. The participants were allocated within 1 day from baseline assessment. eCogT participants were provided with personal usernames and passwords to access the program.

Post‐intervention and follow‐up telephone‐based neuropsychological assessments were undertaken at 7–8 and 27 weeks post‐randomization. Online questionnaires were completed after each neuropsychological assessment. If failing to complete the questionnaires within 7 days, participants received a single reminder over the telephone.

### Training program

The customized training program consisted of 12 tasks from the web‐based program Happyneuron Pro© (Scientific Brain Training, Villeurbanne Cedex, France). This was the only closed access cognitive training program in Danish at the time. Only health care professionals could purchase access. No studies utilizing this program have been published with cognition as outcome.

The program consists of several tasks centred on six cognitive domains: attention, processing speed, learning, memory, working memory and problem‐solving. Task selection was based on domains identified in previous studies 15. Each task is structured as a computer game with ten difficulty levels. Every week, the participants trained two cognitive domains with four different tasks. Each domain and the individual tasks were repeated one time during the training period (e.g. the same tasks were used in week 1 and week 4). Tasks were made available to the individual participants on a weekly basis. For a full description of cognitive training schedule, tasks and technical and content issues, refer to Supplementary Material A (Appendix A1–A3).

Initially, task difficulty levels were the same for all participants (level 1) but automatically advanced when two error‐free trials in a row were completed (up to level 10). Feedback about speed and accuracy was generated automatically upon completion of each task and was readily available. Participants were asked to train a minimum of 30 min/day, 5 days/week, for 6 weeks. The program was available to the participants for up to 8 weeks in case of unforeseen events. Telephone and email‐based support were established to help participants with difficulties. No advice on how to ameliorate cognitive deficits was offered in the support function. Training frequency and intensity were monitored, and the participants received a telephone call if they had not trained for four consecutive days. Furthermore, both groups received two phone calls. The first call was 1 week after group allocation. The timing of the second phone call was unplanned but was towards the end of the intervention, unless the participant had failed to log on, in which case they were always contacted. No adverse effects were reported.

### Neuropsychological assessment

All participants were assessed with standardized neuropsychological tests administered in a fixed order. Premorbid intelligence was estimated with the vocabulary test 16. Test selection was based on cognitive profiles reported in previous studies 15 and on what was possible over the telephone. The assessment took approx. 50 min. To reduce risk of cheating, the participants were instructed not to take notes, and assessors registered any signs of cheating (e.g. suspiciously good test performance or scribbling noises).

### Outcomes

#### Primary outcome

The PASAT 17, 18 was the primary outcome and indicator of *far transfer*, that is, transference of knowledge to unrelated untrained tasks. PASAT is a cognitively demanding test of working memory and attentional capacity consisting of 61 randomized audiotaped digits presented at 3‐s intervals. The participants have to continuously add each digit to the one immediately preceding it. This version was preferred to accommodate possible sound delays. Higher scores reflect poorer performance (0–60).

#### Secondary outcome

Self‐reported cognitive function was assessed with the Cognitive Failures Questionnaire (CFQ) 11. CFQ consists of 25 items (score range 0–100) with higher scores indicating poorer self‐rated cognition.

#### Other cognitive outcomes (for description, refer to Supplementary Material A, Appendix A4)

Verbal memory and learning: Rey Auditory Verbal Learning Test 19. Working memory: Digit Span Forwards, Digit Span Backwards and Digit Ordering 16. Executive function: The Letter Fluency Test 20, The ‘20 Questions Test’ 21 and Cognitive Estimation Task 22.

#### Questionnaires

Demographic and clinical variables: assessed with a short questionnaire. Depression: Beck Depression Inventory, second edition 23. Somatization, illness worrying and conviction: Whitely‐7 24. Anxiety: SCL‐ANX4 from the Symptoms Checklist‐92 25. Both Whitely‐7 and SCL‐ANX4 are subscales from the Common Mental Disorders Screening Questionnaire 26. Self‐reported benefit: Post‐intervention (T2) eCogT participants rated whether the programme had improved their memory and concentration (‘After completing the program, taken together, do you think that the program had a good effect on your memory and concentration?’) on a Likert scale from 0 (not at all) to 4 (to a very great extent). The questionnaires have not yet been validated for online use. Questionnaires assessing sleep quality, fatigue, quality of life, pain, use of health care services, rumination, physical and mental comorbidity were also administered but not included in this publication.

### Statistical analysis

Data were analysed using IBM SPSS Statistics for Macintosh, Version 21.0. (2012; Armonk, NY: IBM Corp). Baseline group differences on demographic, clinical and cognitive variables were examined using independent samples *t*‐tests and chi‐squared tests. Neuropsychological data were converted to *z*‐scores based on means and standard deviations of the WLC to allow for direct comparability between different test versions (e.g. verbal fluency on F, A or N). Mixed linear models (MLMs) were used to compare groups over time. MLM tolerates missing values, and model parameters can be specified as random. The data were hierarchically arranged in two levels, with time (level 1) nested within individuals (level 2). Fixed effects were specified for intercept, time (T1, T2 and T3), group (intervention or waitlist) and time × group interaction. An intervention effect was indicated by a significant time × group interaction, that is, a between‐group difference in changes across the whole time span. All models were based on the intention‐to‐treat sample (*n* = 157), where participants appear with all completed observations and estimated with the maximum likelihood method. Models included a random intercept and repeated effect if it improved the model fit as evaluated by a change in the −2 log likelihood fit statistics 27. Effect sizes were expressed as Cohen's *d*, with 0.2, 0.5 and 0.8 considered as small, medium and large effect size, respectively 28. Cohen's *d* was derived from the *F*‐test and calculated as *d* = 2*√(*F* / df) 29.

## Results

There were no statistically significant baseline differences between eCogT and WLC (refer to Table 1) on any demographic or clinical variables.

**Table 1:** Demographic and clinical characteristics

	Intervention group (eCogT) (*n* = 94)	Waitlist control group (WLC) (*n* = 63)	
	M (SD) or n (%)	M (SD) or *n* (%)	t‐test *X^2^*
Demographic variables:			
Age, M (SD)	54.98 (8.51)	54.56 (8.74)	*t* = 0.30, *p* = 0.76
Education			
Municipal primary and lower secondary school, incl. apprenticeships	21 (22%)	17 (28%)	
Short (<3 years)	21 (22%)	13 (21%)	
Medium (3–4 years)	32 (35%)	20 (33%)	
Long (+5 years)	20 (21%)	11 (18%)	*X^2^* = 0.68, *p* = 0.88
Employment status			
Employed full time	26 (29%)	14 (24%)	
Employed part time	26 (28%)	15 (26%)	
On sick leave	7 (8%)	5 (8%)	
Retired	23 (25%)	15 (26%)	
Other	9 (10%)	9 (16%)	*X^2^* = 1.33, *p* = 0.86
Marital status			
Married/cohabiting	70 (75%)	48 (76%)	
Single/separated/widowed/divorced	24 (26%)	15 (24%)	*X^2^* = 0.60, *p* = 0.81
Premorbid functioning vocabulary^b^	10.48 (2.04)	10.46 (1.98)	*t* = 0.06, *p* = 0.96
Clinical variables:			
Years since diagnosis, M (SD)	4.74 (1.54)	4.44 (2.18)	*t* = 1.01, *p* = 0.31
Surgery:			
Lumpectomy	54 (57%)	32 (51%)	
Mastectomy	38 (40%)	31 (49%)	*X^2^* = 2.31, *p* = 0.32
Treatment:			
Chemotherapy	76 (81%)	54 (86%)	*X^2^* = 0.33, *p* = 0.57^a^
Radiotherapy	80 (85%)	54 (86%)	*X^2^* = 0.00, *p* = 1.00^a^
Hormonal therapy	65 (69%)	44 (70%)	*X^2^* = 0.00, *p* = 1.00 a
Nodal status			
0	39 (43%)	21 (36%)	
1–3	33 (37%)	24 (41%)	
>3	18 (20%)	13 (22%)	*X^2^* = 0.74, *p* = 0.69
Breast cancer metastasis	25 (27%)	21 (34%)	*X^2^* = 0.92, *p* = 0.63^a^

a Chi‐squared test with Yates continuity correction.

b Age‐adjusted scale scores (16).

There were no statistically significant baseline differences between eCogT and WLC on any of the neuropsychological tests or CFQ, depression or anxiety (for raw scores, refer to Table 2). However, eCogT had statistically significant higher scores than WLC on Whiteley‐7 at baseline (*t* = 2.60, *p* = 0.01).

**Table 2:** Raw score‐based descriptives, waitlist control group‐based *z*‐score conversions and MLM results for primary and secondary outcomes

	Baseline	Post‐intervention	Follow‐up	Time × group interaction effect
	WLC (*N* = 63) eCogT (*N* = 94) M (SD)	WLC (*N* = 59) eCogT (*N* = 77) M (SD)	WLC (*N* = 57) eCogT (*N* = 72) M (SD)	*F*; *p* (day)
Primary outcome				
PASAT				
WLC	19.02 (9.88)	15.98 (11.54)	16.04 (10.66)	
eCogT	20.74 (12.37)	17.14 (12.66)	15.15 (10.90)	
eCogT‐*z*	−0.175 (1.25)	−0.101 (1.10)	0.083 (1.02)	1.1; 0.334 (0.15)
Secondary outcomes				
CFQ raw scores				
WLC	46.38 (14.32)	38.63 (13.55)	38.79 (12.54)	
eCogT	44.97 (15.03)	36.62 (12.65)	35.81 (12.95)	0.2; 0.814 (0.07)
RAVLT total score trial I–V^a^				
WLC	48.59 (8.94)	49.33 (8.86)	51.96 (9.52)	
eCogT	48.23 (9.35)	50.94 (8.55)	54.68 (8.91)	
eCogT‐*z*	−0.035 (1.05)	0.018 (0.97)	0.286 (0.94)	3.2; 0.043 (0.22)^*^
RAVLT recall^a^				
WLC	10.00 (2.54)	10.81 (2.44)	11.32 (2.49)	
eCogT	10.10 (2.97)	10.96 (2.84)	11.76 (2.39)	
eCogT‐*z*	0.038 (1.17)	0.062 (1.17)	0.178 (0.96)	0.6; 0.520 (0.10)
Digit Span Forwards^b^				
WLC	8.10 (1.57)	8.25 (1.90)	8.11 (1.67)	
eCogT	8.47 (2.06)	8.86 (2.12)	9.04 (2.08)	
eCogT‐*z*	0.234 (1.31)	0.320 (1.12)	0.560 (1.25)	1.6; 0.207 (0.18)
Digit Span Backwards^b^				
WLC	7.68 (1.76)	8.02 (1.79)	7.96 (1.96)	
eCogT	7.80 (1.67)	8.94 (2.05)	8.71 (1.95)	
eCogT‐*z*	0.067 (0.95)	0.512 (1.13)	0.381 (0.99)	3.3; 0.040 (0.22)^*^
Digit Span Ordering^b^				
WLC	7.89 (1.90)	8.81 (1.88)	8.28 (1.84)	
eCogT	8.18 (1.90)	8.94 (1.95)	8.90 (1.99)	
eCogT‐*z*	0.153 (0.99)	0.067 (1.04)	0.338 (1.08)	1.0; 0.353 (0.12)
Letter fluency				
WLC^c^	14.44 (4.88)	10.68 (4.66)	10.89 (4.92)	
eCogT	14.85 (6.14)	11.49 (5.43)	12.21 (4.96)	
eCogT‐*z*	0.084 (1.26)	0.174 (1.64)	0.268 (1.01)	0.4; 0.697 (0.10)
CET				
WLC^d^	3.20 (2.29)	2.56 (1.76)	6.23 (2.54)	
eCogT	2.72 (2.30)	2.51 (1.58)	5.79 (2.46)	
eCogT‐*z*	0.211 (1.01)	0.028 (0.90)	0.172 (0.97)	0.3; 0.709 (0.07)
20 total^a^				
WLC	12.95 (3.54)	14.64 (4.39)	13.98 (4.74)	
eCogT	13.58 (4.93)	14.16 (4.17)	13.76 (4.62)	
eCogT‐*z*	−0.177 (1.39)	0.111 (0.95)	0.046 (0.97)	0.7; 0.495 (0.10)
20 abstract^a^				
WLC	14.11 (5.86)	15.19 (6.02)	15.80 (6.10)	
eCogT	13.71 (6.12)	15.71 (6.29)	15.32 (6.18)	
eCogT‐*z*	−0.068 (1.04)	0.088 (1.05)	−0.077 (1.01)	0.5; 0.613 (0.09)
BDI^a^				
WLC	15.00 (8.70)	13.77 (8.90)	12.39 (7.99)	
eCogT	13.02 (8.52)	10.32 (8.86)	11.04 (9.00)	2.7; 0.067 (0.20)
Anxiety^a^				
WLC	3.22 (2.78)	3.34 (3.31)	2.89 (2.47)	
eCogT	2.51 (2.66)	2.18 (2.38)	2.25 (2.47)	0.4; 0.683 (0.07)
Somatization (Whitely‐7)^a^				
WLC	6.21 (5.52)	6.00 (4.16)	5.86 (4.83)	
eCogT	4.06 (4.27)	4.11 (4.45)	4.07 (4.47)	0.1; 0.933 (0.03)

20 total, 20 questions, total number of questions posed; 20 abstract, 20 questions, abstraction level of first question; CET, cognitive estimation task; eCogT‐*z*, *z*‐scores for the eCogT group.

a Raw score.

b Maximum span length.

c Number of correct responses for the letters ‘F’ (T1), ‘N’ (T2) and ‘A’ (T3).

d Raw score (maximum = 5).

The eCogT group engaged in cognitive training for an average of 16.78 h (SD = 7.97 h, range 1–40 h) over the intervention period. Thereof, 78% trained more than 10 h, whilst 65% used the programme for 15 h or more. No group × time interaction (*F*(2, 198.4) = 1.1, *p* = 0.334) was detected for the primary outcome (i.e. PASAT; refer to Table 2). Two statistically significant results were found for the other outcomes. eCogT exhibited an increase in verbal learning (Rey Auditory Verbal Learning Test (RAVLT)) compared with WLC (*F*(2, 272.1) = 3.2, *p* = 0.043) (refer to figure, Appendix A5), and a small effect was found on Digit Span Backwards (*F*(2, 272.6) = 3.3, *p* = 0.040) (refer to figure, Appendix A6). No other statistically significant group × time interactions were found.

Post‐intervention, 5.2% of the eCogT group expressed that the programme did not have an positive effect on their memory and concentration, 37.7% reported it had a limited effect, 35.1% reported that it had some effect, 20.8% reported that it had an effect to a great extent, and 1.3% reported that it had an effect to a very great extent. Taken together, 42.9% reported none or limited effect whilst 57.1% stated having had some or great effect.

## Conclusion

The present study is the largest randomized controlled trial to date of eCogT for breast cancer survivors. No significant improvement was found in the intervention group compared with the control group for the primary outcome PASAT. Furthermore, although the majority of participants reported having had some or a great effect of the programme on their memory and concentration, we found no statistically significant reduction in CFQ scores. Cognitive training did result in improvements of verbal learning and on a test of working memory. These effects were observed at 5‐month follow‐up, suggesting long‐term effects.

One explanation for the limited results could be that the participants had insufficient cognitive impairment at baseline. Only 24% (28% eCogT and 19% WLC) scored below the education‐corrected fifth percentile cut‐off for PASAT at baseline 30 which may have reduced our ability to detect change (i.e. a ceiling effect). The cut‐off on PASAT, however, is based on a small American sample (*n* = 101) that may not generalize to Danish women 30. Notwithstanding, even if scores were in the unimpaired range, the participants might have experienced a reduction from higher premorbid levels.

Improved verbal learning and Backwards Digit Span after cognitive training indicates working memory improvements. However, these appear limited and were not observed for tests with greater cognitive load (e.g. PASAT). Whereas previous research on cognitive training for breast cancer survivors has focused on training specific cognitive functions, for example, executive functioning 10, memory and processing speed 31, memory and attention 32, we targeted several functions simultaneously, reflecting the array of cognitive complaints reported by breast cancer survivors 15. The improvement of working memory may reflect that most training tasks require working memory, whereby it has been trained the most. Conversely, the findings may reflect that the study design primarily allows detection of far transfer effects. A concern in web‐based training is whether improvements are seen in functions that are not trained 9, that is, whether there is *transfer* of knowledge from a trained task to a related but untrained task and also whether *far transfer* occurs (i.e. improvements on unrelated untrained tasks). In the present study, we solely presented training tasks visually, while the cognitive assessments were delivered auditorily. Furthermore, only one training exercise strongly resembled the cognitive tests (RAVLT). These issues raise the question whether the discrepancy between visual training and verbal assessment means that it would mainly be possible to discover far transfer effects. Future studies could assess the effects of cross‐modality training and assessment on transfer of training gains.

Previous clinic‐based interventions for breast cancer survivors have also only found improvements on single measures of cognition and not generalized improvements within or across several domains. Thus, five randomized controlled studies (RCTs) have explored psychological interventions to alleviate cognitive complaints in cancer survivors 1. Thereof, three report improvements in only one objectively assessed cognitive function out of a large test battery: digit span improvements (*d* = 0.81; treatment group: *n* = 12) 33, psychomotor speed improvements (*d* = 0.67; treatment group: *n* = 16) 34 and learning on a verbal memory test (*d* = 0.63; treatment group: *n* = 19) 32. Furthermore, two of these studies found improved subjective cognitive function assessed with the Functional Assessment of Cancer Therapy‐Cognitive Function questionnaire 33, 34. The fourth RCT compared processing speed training (*n* = 30) and memory training (*n* = 29) with a waitlist condition and found significant improvements on a composite memory score in both intervention groups at 2‐month follow‐up (*d* = 0.55–0.72) 31. They also reported improved processing speed on a composite score after processing speed training at 2‐month follow‐up (*d* = 0.67) 31. A fifth RCT by Kesler *et al.* (2013) of web‐based training of executive function found improvements of executive functions on three cognitive tests immediately post‐intervention (*d* = 0.58–0.87) 10. However, two of the three outcome measures included in their study were conceptually similar to the training tasks, which may have boosted the intervention effect. Furthermore, in the present study, participants trained for 6 weeks (total requested training time: 15 h) whilst the training period in the study by Kesler *et al.* (2013) was 12 weeks (total requested training time of 24 h) 10. Unfortunately, it is not possible to compare adherence in the present study to the study by Kesler *et al.* (2013) as they do not report the number of training hours. In the present study, we found variability in adherence with an average of 78% of participants training 10 h or more and 65% training the specified 15 h or more. Future research should establish an optimal training period both in terms of daily use and number of weeks.

Some limitations of the present study should be noted. First, only a subset of the neuropsychological tests used has been validated for telephone‐based administration 35. Generally, studies comparing face‐to‐face and telephone‐based assessment find no significant effect of assessment mode 35, 36. Furthermore, some subtests included in the present study resemble subtests included in the Brief Test of Adult Cognition by Telephone battery, which are telephone‐delivered tests that have been administered in a large sample (*n* = 4268) 37 and validated against face‐to‐face assessment (*n* = 84) 36. Hence, there are indications of the usability of telephone‐based assessment. Another potential issue with telephone‐based assessment is risk of cheating. We found no indications of cheating, but this cannot be ascertained without further data (e.g. concurrent webcam access). Second, the questionnaires have not been validated for online administration. Generally, previous studies conclude that there are no differences between online and paper‐and‐pencil questionnaires on psychometric properties 38, particularly when sampling is controlled 39. Third, it is possible that the observed improvements are secondary benefits through reduction of psychological distress from participating in a study rather than cognitive training *per se*. A near‐significant (*p* = 0.067) reduction in depressive symptoms was observed, and it is possible that a more sensitive measure would have captured reductions in distress. Fourth, the present design did not allow for concealment of conditions nor did it include an active control group. Finally, the effect of training on everyday functioning was not assessed.

In conclusion, the intervention may be applied at a low cost and without risk of adverse effects. It was well received, with the majority estimating a beneficial effect on memory and concentration. The effects on objectively assessed cognition, however, are limited to a measure of working memory and verbal learning with small but positive and durable effects.

## Conflict of interest

None

## Supporting information

Supporting info item
