# Supplementary material for: Web‐based cognitive training for breast cancer survivors with cognitive complaints—a randomized controlled trial
Source: Psychooncology. 2016 Jan 13;25(11):1293–300. doi: 10.1002/pon.4058 (PMC5111748; doi:10.1002/pon.4058)
Supplement: Supplementary file 1 — Supporting info item [file PON-25-1293-s001.docx]

**ARTICLE TITLE:** Web-based cognitive training for breast cancer survivors with cognitive complaints – a randomized controlled trial.

**AUTHORS**: Damholdt M.F., Mehlsen M.Y^.^, O’Toole M.S., Andreasen R.K., Pedersen A.D., Zachariae R.

**Table of content**

Appendix A 1: Cognitive training schedule 2

Appendix A 2: Description of cognitive tasks in the training program 3

Appendix A 3: Technical and content issues during the trial 8

Appendix A 4: Table of neuropsychological tests 9

Appendix A 6 (fig.3) Mean -z score digit span backwards for WLC and eCogT groups at T1-T3 13

References 14

Appendix A 1: Cognitive training schedule

| **Attention and processing speed**  *Week 1 and week 4* | **Memory and learning**  *Week 2 and week 5* | **Working memory and problem-solving**  *Week 3 and week 6* |
| --- | --- | --- |
| **Abilities trained:**   - focused attention - vigilance - response time and accuracy | **Abilities trained:**   - verbal and visual memory - learning strategies | **Abilities trained:**   - Maintain and process perceptions simultaneously - Verbal and visual problem-solving |
| **Exercises:**  Day 1:  “Under pressure”  “Surveillance”  Day 2:  “Two things at a time”  “Complex figures”  Day 3:  “Under pressure”  “Two things at a time”  Day 4:  “Complex figures”  “Surveillance”  Day 5:  “Under pressure”  “Complex figures” | **Exercises:**  Day 1:  “Memory as an elephant”  “Restaurant”  Day 2:  “Coat of arms”  “The trip”  Day 3:  “Memory as an elephant”  “Coat of arms”  Day 4:  “The trip”  “Restaurant”  Day 5:  “Memory as an elephant”  “The trip” | **Exercises:**  Day 1:  “Right place, right time”  “Secret archives”  Day 2:  “Star gazing”  “Basketball”  Day 3:  “Right place, right time”  “Star gazing”  Day 4:  “Secret archives”  “Basketball”  Day 5:  “Right place, right time”  “Basketball” |

Appendix A 2: Description of cognitive tasks in the training program

| **Task name** | **Task description** | **Sensory stimulation and feedback** | **Determinants of difficulty level^a^** |
| --- | --- | --- | --- |
| “Under pressure” | Three stimuli are shown in turn different places on screen. The participant has to indicate if a red circle is shown above or below a black -*x* by pressing the keyboards’ arrow-key up or down. | Visual introduction text and three different visual stimuli (red circle, black cross and a letter). Auditory stimulation at start-up. Feedback: Two visual scales providing information on precision (% correct) and response time (seconds). The participants own and the correct answers are displayed. | Number of stimuli (10 or 20), stimuli-display time, spatial distance between stimuli- and distraction item. |
| “Surveillance” | The participants have to find a target-symbol in a grid-pattern filled with different, nearly identical symbols. | Introduction text, different shapes, and symbols. Auditory feedback depending on whether the participant has answered correctly. Feedback: Two visual scales providing information on precision (% correct) and response time (seconds). | Varying size of grid-pattern (4x4 to 8x16) and varying distractor-items. |
| “Two things at a time” | The participant has to simultaneously press a certain key when presented with a given symbol and sort words presented auditorily into categories. | Introduction text, different categories and symbols. Auditory presented categories (e.g. animals, clothes). Auditory feedback depending on whether the participant has answered correctly. Feedback: Two visual scales providing information on precision (% correct) and reaction time (milliseconds). | Varying number of task-series (4-10), stimuli-display time, table size (2-9 grids), number of auditory categories (2-4) |
| “Complex figures” | The participant has to analyse a target-figure that consists of several individual elements. These elements have to be identified from a selection of distractor- elements. | Introduction text and figures. Auditory feedback depending on whether the participant has answered correctly. Feedback: Text providing information on precision (% correct) and response time (seconds) | Varying figure-types (figures, geometrical shapes or amoebas), varying number of elements in the target-figure (2-3), simultaneous or delayed display of target-figure and elements. |
| “Memory like an elephant” | The participant has to learn different word-lists. Then the first learned wordlist has to be recognized amongst a number of distractor words | Introduction text. Auditory stimulation at start-up.  Feedback: Text providing information on precision (% correct) and response time (seconds). The participants own and the correct answers are displayed. | Varying number of words to be learned (6-20), variation in the semantic similarity between words, varying display time. |
| “Restaurant” | The participant has to pretend being a waiter taking food- and drink orders from guests in a restaurant. | Introduction text. Text showing the name of guests and their orders. Auditory feedback depending on whether the participant has answered correctly. Feedback: Text providing information on precision (% correct) and response time (seconds). The participants own and the correct answers are displayed. | Varying number of guests (2-4), different placement at the table from when they order till they get served, varying number of courses (2-4), number of distractor-courses (i.e. courses no one has ordered), distractions from order is taken till food is served (e.g. having to inform on calorie amount in the food). |
| “Coat of arms” | The participant has to remember a coat of arms and all its elements (i.e. colour, shape, patterns and different symbols). A distraction task is presented before recall of the coat of arms with all its components. | Introduction text and coat of arms with different characteristics. Auditory feedback depending on whether the participant has answered correctly. Feedback: two visual scales providing information on precision (% correct) and response time (seconds) during learning. Furthermore, the participant’s own and the correct answers are displayed. | Varying pattern complexity (composition of colour), number of symbols (0-3), amount of time for learning (30-60 seconds), type of distraction task (sorting numbers numerically V. sorting letters alphabetically). |
| “The trip” | The task consists of two parts: 1) remembering names and locations of different attractions on a city map, 2) placing the attractions correctly on a map in the order they were presented. | Introduction text and different icons. Auditory feedback depending on whether the participant has answered correctly.  Feedback: two visual scales providing information on precision (% correct) and response time (seconds) | Varying number of attractions (3-7), varying time for learning (8-10 seconds). |
| “Right place, right time” | The participant has to observe symbols presented on the right side of the screen and then identify which symbols are not present on the left side of the screen. | Introduction text, different symbols, indicator of how long the participant has to identify the symbol. Auditory feedback depending on whether the participant has answered correctly.  Feedback: two visual scales providing information on precision (% correct) and response time (seconds), which is also described in text. | Varying number of symbol (5-10), type of symbol (Egyptian, Mayan, Thai or runic letters). |
| “Secret archives” | Words drop from the top of the screen and the participant has to place them in archiving-drawers corresponding to word categories. Some of the drawers have labels with category names on them whilst others do not. | Introduction text, words and drawers. Auditory feedback depending on whether the participant has answered correctly.  Feedback: Three visual scales providing information on precision (% correct), response time (seconds) and number of mistakes. Furthermore, the participants own and the correct answers are displayed. | Varying number of drawers (2-6) and different number of drawers without labels. |
| “Stargazing” | The participants have to fill out a star-shaped crossword puzzle with words listed to the right side of the screen. | Introduction text. Visual presentation of different instruments. Auditory feedback depending on whether the participant has answered correctly. Two visual scales providing information on precision (% correct), and response time (seconds), which is also described in text. Furthermore, the participants own and the correct answers are displayed | Varying number of target-words (6-12), varying number of distractor words (0-6). Number of cues visible on the star (0-1 letter or a word is visible in the beginning). |
| “Basketball” | Two rows of line-drawn basketball nets filled with varying number of coloured basketballs are displayed. The participants have to deduce how many moves are necessary to make the bottom row identical to the top row. | Introduction text. Auditory feedback depending on whether the participant has answered correctly. Feedback: Two visual scales providing information on precision (% correct), and response time (seconds), which is also described in text. Furthermore, the participants own and the correct answers are displayed | Varying number of basketballs (3-5), varying number of moves necessary. |
| **Supplementary exercises** | | | |
| Auditory task | Auditory stimuli are presented from six different instruments. The participants have to discriminate amongst them based on pitch, length of tone and volume. | Auditory presentation of stimuli. Auditory feedback depending on whether the participant has answered correctly. Feedback: Two visual scales providing information on precision (% correct), and response time (seconds), which is also described in text. Furthermore, the participants own and the correct answers are displayed | Varying volume, pitch and tone lenght |
| Birdsong | The participants have to remember the name and songs of different species of birds | Introduction text, visual presentation of different species of birds. Auditory presentation of different birdsongs. . Auditory feedback depending on whether the participant has answered correctly. Information about precision (% correct), and response time (seconds) with regards to bird species and song. | Varying length of stimuli-presentation time (30 seconds, 60 seconds or unlimited), varying number of birds (2-6). |

^a^All tasks have 10 levels that are pre-programmed and adjust to the individuals’ performance

Appendix A 3: Technical and content issues during the trial

| **List of technical issues during the trial** | **Description** |
| --- | --- |
| General issues |  |
|  | The program was not IPAD-compatible and the participants were advised thereof at enrolment. |
|  | The combination of using a MacBook Air and the browser “Safari” resulted in the entire program being displayed in French instead of Danish. The participants were informed on this issue and a “system requirements” paragraph was added as a footnote to all e-mails to the participants. Participants who experienced the problem were advised to use another browser when accessing the program. |
| Issues pertaining individual tasks |  |
| “Under pressure” task | During the first levels a letter/number is presented in the middle of the screen, then the letter *x* and then a red circle (target). On the higher levels (level 9-10) distractor items are introduced (letters). In 1 out of 35 exercises a situation arises in which the distracting stimuli is an *x* whereby two *x*’s are presented after each other (distractor and the one that forewarns the target-stimuli). Nine participants contacted the support-email and complained that the target stimuli had been missing (i.e. the red circle that would normally appear after the black cross). |
| “Birdsong” task | An ornithologist believed that the name of a specific species of owl had been wrongly translated. Verification has as of yet not been obtained. |

Appendix A 4: Table of neuropsychological tests

| Test | Cognitive domain | Assessment  times^1^ | Description |
| --- | --- | --- | --- |
| Other outcomes | | | |
| Rey Auditory Verbal Learning Test (1,2) | Verbal learning and memory | T1, T2, T3 | Verbal memory and learning was assessed with Rey Auditory Verbal Learning Test (1,2), consisting of 5 presentations of a 15-item wordlist. Immediate recall is administered after each presentation and delayed recall after approx. 20 minutes. Different versions (A, B or C) were used at the three assessment times. The following scores were registered: Total acquisition score: sum trial I-V. Retention percentage score: delayed recall/recall trial 5×100. Delayed recall after approx. 20 minuts. |
| Digit Span Forward (3) | Working memory | T1, T2, T3 | The test consists of eight pairs of random number sequences of increasing to be repeated by the participant |
| Digit Span Backwards (3) | Working memory | T1, T2, T3 | The test consists of eight pairs of random number sequences of increasing to be repeated backwards by the participant. |
| Digit Span Ordering (3) | Working memory | T1, T2, T3 | The test consists of random number sequences of increasing length read aloud to the participant who has to repeat them in numerical order. |
| Letter Fluency (4) | Executive functioning | T1, T2, T3 | The Letter Fluency Test requires the participant to name as many words as possible starting with the letter “F” (T1), “N” (T2), or “A” (T3) within a 60-sec. time limit |
| The Cognitive Estimation Task (CET; (5)) | Executive functioning | T1, T2, T3 | 15 items (five at each assessment time) from the Danish CET were administered, consisting of questions that are not immediately answerable, but for which an answer can be deduced based on general knowledge. Higher scores reflect poorer performance. It was administered with Qualtrics Software © (2013; Qualtrics, Provo, UT, USA). Each question had to be answered within 30 sec. to prevent searching the correct answer online. |
| The 20 Questions Test (6) | Executive functioning | T1, T2, T3 | The “20 Questions Test” consists of 30 line-drawn pictures on an A4 sheet of paper posted to the participant in a sealed envelope with instructions not to open it before the assessment. By asking yes/no questions the participant has to deduce which picture the examiner is thinking of. Two different subtests were administered at each assessment point and two sub-scores registered: total number of questions asked and abstraction score of the first question asked (lowest number of objects that could be eliminated by the participant’s first question |
| Vocabulary (3) | Premorbid Intelligence | T1 | The participants are required to define 30 words of increasing difficulty. |

^1^ Baseline (T1), Post-intervention (T2) and follow-up (T3)


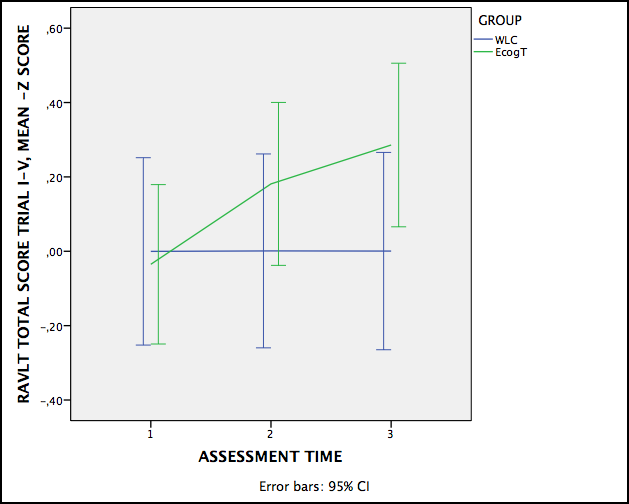


Appendix A 5 (fig.2) RAVLT total score trial I-V, mean -z score for WLC and eCogT at T1-T3

Appendix A 6 (fig.3) Mean -z score digit span backwards for WLC and eCogT groups at T1-T3


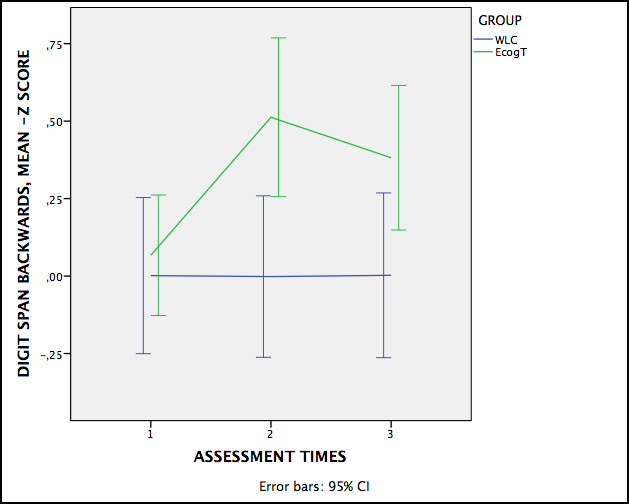


REFERENCES

1. Rey A. L’examen clinique en psychologie. Paris: Presses Universitaires de France; 1964.

2. Schmidt M. Rey auditory verbal learning test: A handbook. Los Angeles CA: Western Psychological Services; 1996.

3. Wechsler D. Wechsler adult intelligence scale–Fourth Edition (WAIS–IV). San Antonio TX NCS Pearson [Internet]. 2008 [cited 2015 Apr 24]; Available from: http://www.statisticssolutions.com/academic-solutions/resources/directory-of-survey-instruments/wechsler-adult-intelligence-scale-fourth-edition-wais-iv/

4. Benton AL, Hamsher K. Multilingual aphasia examination (2nd ed.). Iowa City, IA: AJA Associates.; 1976.

5. Shallice T, Evans ME. The involvement of the frontal lobes in cognitive estimation. Cortex. 1978;14(2):294–303.

6. Delis DC, Kaplan E, Kramer JH. Delis-Kaplan executive function system (D-KEFS). Psychological Corporation; 2001.
